# The effectiveness of various gargle formulations and salt water against SARS-CoV-2

**DOI:** 10.1038/s41598-021-99866-w

**Published:** 2021-10-15

**Authors:** Vunjia Tiong, Pouya Hassandarvish, Sazaly Abu Bakar, Nurul Azmawati Mohamed, Wan Shahida Wan Sulaiman, Nizam Baharom, Farishah Nur Abdul Samad, Ilina Isahak

**Affiliations:** 1 Tropical Infectious Diseases Research and Education Centre (TIDREC), University of Malaya, Kuala Lumpur, Malaysia; 2 Faculty of Medicine and Health Sciences, Universiti Sains Islam Malaysia, Persiaran Ilmu, Bandar Baru Nilai, 78000 Nilai, Negeri Sembilan Malaysia; 3 Ministry of Health, Putrajaya, Malaysia

**Keywords:** Microbiology, Medical research

## Abstract

The COVID-19 is difficult to contain due to its high transmissibility rate and a long incubation period of 5 to 14 days. Moreover, more than half of the infected patients were young and asymptomatic. Virus transmission through asymptomatic patients is a major challenge to disease containment. Due to limited treatment options, preventive measures play major role in controlling the disease spread. Gargling with antiseptic formulation may have potential role in eliminating the virus in the throat. Four commercially available mouthwash/gargle formulations were tested for virucidal activity against SARS-CoV-2 in both clean (0.3 g/l BSA) and dirty (0.3 g/l BSA + 3 mL/L human erythrocytes) conditions at time points 30 and 60 s. The virus was isolated and propagated in Vero E6 cells. The cytotoxicity of the products to the Vero E6 was evaluated by kill time assay based on the European Standard EN14476:2013/FprA1:2015 protocol. Virus titres were calculated as 50% tissue culture infectious dose (TCID50/mL) using the Spearman-Karber method. A reduction in virus titer of 4 log_10_ corresponds to an inactivation of ≥ 99.99%. Formulations with cetylperidinium chloride, chlorhexidine and hexitidine achieved > 4 log10 reduction in viral titres when exposed within 30 s under both clean and dirty conditions. Thymol formulations achieved only 0.5 log_10_ reduction in viral titres. In addition, salt water was not proven effective. Gargle formulations with cetylperidinium chloride, chlorhexidine and hexetidine have great potential in reducing SAR-CoV-2 at the source of entry into the body, thus minimizing risk of transmission of COVID-19.

## Introduction

Since its first discovery in December 2019, coronavirus disease 2019 (COVID-19) has affected more than 200 million people and caused more than 4 million deaths worldwide^1^. Severe Acute Respiratory Syndrome Coronavirus-2 (SARS-CoV-2) has been implicated as the causative agent of COVID-19 based on genetic analysis of the virus from the first few cases detected in Wuhan, China^2^. SARS-CoV-2 is an envelope, non-segmented, positive sense RNA virus identified as a novel beta coronavirus after SARS-CoV and MERS-CoV^3^. The virus spreads via respiratory droplets when infected individuals sneeze or cough or indirectly through objects contaminated with respiratory droplets. The pathogenesis begins when SARS-CoV-2 attacks the nasal cavity, nasopharynx, oropharynx and eye mucus membranes. The virus replicates in the nose and throat, as evidenced by the high viral load at the early stage of infection, at the beginning of symptoms^4^.

The COVID-19 is difficult to contain due to high transmissibility rate and a long incubation period of 5 to 14 days^5^. Moreover, more than half of the infected patients were young and asymptomatic^6^. Virus transmission through asymptomatic patients is a major challenge to disease containment. To date, there is no effective specific antiviral for SARS-CoV-2. Even though remdesivir, hydroxychloroquine and lopinavir-ritonavir combination has been tested clinically, many trials suggest that these drugs are not completely effective for the treatment of COVID-19^7^. Due to limited treatment options for COVID-19, preventive measures play a major role in controlling the disease spread. The World Health Organization recommends four measures which are regular hand washing, mask wearing, physical distancing and cough etiquette^8^. In addition, many countries have adopted movement control orders or lockdown to enhance the measures^9,10^. COVID-19 vaccine that has been introduced in late 2020 has been shown to effectively reduce the severity of the disease^11^, however the number of cases and deaths are still growing worldwide^1^. Therefore, more research has to be done to explore new treatment and preventive strategies in order to curb the spread of the disease.

Saliva samples of COVID-19 positive patients have been shown to contain live viruses that may cause viral transmission, putting healthcare workers who work in close proximity with the face and oral cavities, like dental professionals, at high risk of acquiring infection^12^. Moreover, usage of ultrasonic and high-speed handpiece instruments generates high amount of blood and salivary aerosols. Indirect contact with contaminated surfaces or instruments also promotes cross-infection^13^. The American Dental Association (ADA) and Centre of Disease Control and Prevention (CDC) have urgently called for dental professionals to use the highest level of infection control since the beginning of this novel coronavirus outbreak. Pre-procedural mouth rinsing (PPMR) has been advocated as a measure to prevent saliva-mediated disease transmission in a dental setting^14^. In accordance with this, the Ministry of Health Malaysia has recommended in its guideline the use of mouthwash containing 1.0–1.5% hydrogen peroxide or 0.2–1.0% povidone iodine prior to dental examination and treatments^15^.

The effectiveness of povidone-iodine (PVP-I) against SARS-CoV-2 has been proven by various studies. An in vivo study among four COVID-19 positive patients suggested that povidone iodine gargling could reduce the saliva viral load of SARS-CoV-2 for at least three hours after gargling^16^. Meanwhile, an in vitro study showed that povidone-iodine mouthwash/gargle/ 1% achieved > 5 log10 reduction in the SARS-CoV-2 titre after at least 15 s of treatment^17^. However, PVP-I is not recommended for those with active thyroid disease, children, pregnancy, patients undergoing radioactive iodine therapy and those with anaphylactic allergy to iodine^18^. It is also costly and not widely available in the market; these factors are important in encouraging uptake of simple preventive measures that the general public can use. Therefore, there is a need to explore other commercial mouthwash formulations, to provide alternatives for the healthcare workers and the public to prevent COVID-19. In this study, we tested four commercially available mouthwash/gargle formulations and salt water against SARS-CoV-2.

## Material and methods

### Cells and viruses

Vero E6 cells were cultured in Dulbecco's modified Eagle's medium (DMEM) (Gibco, Grand Island, NY, USA) added with 10% fetal bovine serum (FBS) and kept inside CO_2_ incubator with 37ºC. Vero E6 was used for virus propagation and titration. TCID50 method used to determine virus titration and it expressed in TCID50/mL. SARS-CoV-2 virus was isolated from nasopharyngeal/oropharyngeal (NP/OP) swab sample from SARS-CoV-2-positive individual. The NP/OP swabs were collected in commercially available virus transport medium which contains antimicrobial agents as stated in the data sheet. Patient characteristics were not recorded for the propagation of the virus. Briefly, the NP/OP sample was inoculated into Vero E6 cells and incubated until visible cytopathic effect. To prevent microbial growth during the propagation of the virus, antibiotics and antimycotics were added to the culture medium during the propagation. The clarified supernatant containing SARS-CoV-2 was harvested as virus stock, titrated by plaque assay, and stored at − 80 °C until further use. The virus stocks prepared (SARS-COV-2/MY/UM/6-3; TIDREC) were confirmed by qPCR prior to use for subsequent experiments (data not shown). All procedures that required handling of live virus was performed in the Biosafety Level 3 (BSL3) laboratory at the Tropical Infectious Diseases Research & Education Centre (TIDREC), Universiti Malaya.

### Mouthwash/gargle formulations

Four commercially available mouthwash/gargle formulations and laboratory prepared salt water (2% sodium chloride) were used in this study as shown in Table 1.

**Table 1:** List of mouthwash/gargle formulations.

Product	Trade name	Active ingredients
A	Oradex®	0.12% chlorhexidine digluconate
B	Colgate Plax® Fruity Fresh	0.075% Cetylperidinium chloride, 0.05% Sodium fluoride
C	Thymol® Mouthwash by Xepa	0.05% Thymol
D	Bactidol®	0.1% Hexetidine 9% Ethanol
E	Salt water	2% (0.34 M) Sodium chloride

### Viral kill time assay

The gargle formulations were tested against SARS-CoV-2 in accordance to the European Standard EN14476:2013/FprA1:2015 protocol as described by Eggers et al.^19^. Briefly, the products were tested under 2 different conditions (interfering substance): clean (0.3 g/l BSA) and dirty (0.3 g/l BSA + 3 mL/L human erythrocytes. The test assay comprised of 100 µl of interfering substance, 100 µl of virus suspension at concentration of 5 × 10^5^ TCID50/mL and 800 µl of test product (mouthwash/gargle). After the specified contact time (30 s and 60 s), the virucidal activity of the product was suppressed by a dilution tenfold dilution in ice cold media (DMEM + 2% FBS). Serial dilutions were then prepared and added to Vero E6 cells to determine the TCID50/mL. Virus controls for this test was cell culture medium water in place of test product for both dirty and clean conditions. The cells were incubated for 96 h till obvious CPE developed. 4% paraformaldehyde and 0.5% crystal violet were used to fix and stain the infected cells, respectively. The virus titers were determined using the Spearman-Karber method and expressed TCID50/ml. The virucidal activity was determined by the difference of the logarithmic titer of the virus control minus the logarithmic titer of the test virus (Δ log10 TCID50/ml). A reduction in virus titer of ≥ 4 log10 corresponded to an inactivation of ≥ 99.99% virus.

#### Formulations suppression assay

The suppression assay was performed to ensure the mouthwashes virucidal effect after neutralization don’t show any effectivity. Briefly, all mouthwash formulations were diluted 10 × inside DMEM with 2% FBS. The 10 × diluted formulations were added inside the tube conation 100 µl of SARS-CoV-2 virus and allowed for 1 min of contact time. The mixtures were suppressed by performing 10 × serial dilution after 1 min contact time and 100 µl of different dilution were added into the cells. The cells were compared to virus control and present of virus cytopathic effect is sign of the formulations don’t show any effectivity after mouthwashes suppression.

### Calculation of the virucidal activity of the products

The virucidal activity was determined by the difference of the logarithmic titre of the virus control minus the logarithmic titre of the test virus (Delta log10 TCID50/ml).

## Results

### Virucidal activity of mouthwash/gargle formulations

The SARS-CoV-2 titre in the control-treated samples under clean and dirty conditions were both at 5.4 × 10^5^ TCID50/ml. Cetylperidinium chloride and hexetidine achieved > 5 log10 reduction in viral titers when exposed within 30 s under both clean and dirty conditions. Chlorhexidine digluconate achieved 4 log10 reduction in viral titers within 30 s under both clean and dirty conditions. Salt water and thymol did not show any significant reduction in viral load. (Table 2).

**Table 2:** Virucidal activity of mouthwash/gargle formulations against SARS-CoV-2.

Product	Trade name	Active ingredients	Virus	Log10 Reduction in viral titers compared to control			
				Clean condition		Dirty condition	
				30 s	60 s	30 s	60 s
A	Oradex®	0.12% chlorhexidine digluconate	SARS-CoV-2	4.00	4.00	4.00	4.00
B	Colgate Plax® Fruity Fresh	0.075% Cetylperidinium chloride, 0.05% Sodium flouride		5.00	5.00	5.00	5.00
C	Thymol® Mouthwash by Xepa	0.05% Thymol		0.5	0.75	0.5	0.5
D	Bactidol®	0.1% Hexetidine 9% Ethanol		5.00	5.00	5.00	5.00
E	Salt water	2% (0.34 M) Sodium chloride		0.00	0.00	0.00	0.00

Results from the suppression assay showed no difference in viral titres compared to controls (Table 3). This suggested that addition of cold media and serial dilution effectively suppressed the product activity, resulting in no reduction of the viral titre.

**Table 3:** Suppression of product virucidal activity.

Contact time (s)	Interfering substance	Viral Titre [Control] (TCID50/ml)	Viral Titre [After product suppression] (TCID50/ml)	Difference in Viral Titre (TCID50/ml)
60	clean conditions	5.4 × 10^5^	5.4 × 10^5^	0.00
60	dirty conditions	5.4 × 10^5^	5.4 × 10^5^	0.00

## Discussions

This study shows that cetylperidinium chloride and hexetidine-containing mouthwash/gargle were able to decrease > 99.99% of SARS-CoV-2 concentration from the initial level after as early as 30 s exposure. Cetylperidinium chloride is a cationic quaternary ammonium compound that is frequently used as detergents and antiseptics^20^. It causes virus inactivation by disrupting the lipid envelope, destroying the capsid, as well as through its lysosomotropic action^21^. This study showed that cetylperidinium chloride in combination with sodium fluoride was very effective in inactivating SARS-CoV-2 after 30 s of contact time. An earlier study demonstrated its efficacy against different strains of influenza viruses^22^. Due to the mechanism of action, it is believed that cetylperinidium may also act effectively against other enveloped viruses such as respiratory syncytial virus (RSV) and parainfluenza.

Hexetidine, has broad spectrum antibacterial and antifungal activities, particularly against gram-positive bacteria and fungi. Hexetidine destructs bacterial cell walls and inhibit oxidative metabolic reactions (thiamine antagonist). Though the antiviral properties was not well studied, it has been shown that hexetidine attenuated highly virulent influenza virus A/H5N1, pandemic influenza virus A/H1N1, respiratory syncytial virus, and herpes simplex virus type 1 after a short-term exposure^23^. Ethanol was known to exert antibacterial and antiviral effect at a minimum concentration of 40–60%^24^ . Lower concentration of ethanol in combination with hexetidine in this study may further enhance the antiviral effect against SARS-Cov-2 virus.

Chlorhexidine mainly acts on bacteria by disrupting bacterial membrane thus increasing membrane permeability that results in cell death and lysis^25^. It is widely used in dentistry for the management bacteria related conditions such as gingivitis and periodontitis^26^ and in the intensive care setting for the prevention of nosocomial pneumonia^27^. This study found that the virucidal activity of chlorhexidine containing mouthwash against SARS-CoV-2 was slightly lesser than those of cetylperidinium chloride and hexetidine. This result is incongruent with a study which showed log reduction factors ranging from 0.33 to 0.78 only^28^. However, other recent in vitro and in vivo studies showed that chlorhexidine (0.12% to 2% concentration) was effective against SARS-CoV-2^29,30^.

Thymol is naturally found in the oils of thyme (*Thymus vulgaris L., oregano (Origanum vulgare), Satureja thymbra, Thymbra capitata and Monarda fistulosa L*.^31^. Its aqueous extract showed high antiviral activity against HSV-1, HSV-2 and an acyclovir-resistant strain of HSV-1^32^. Thymol in combination with other essential oils had been shown to work effectively against enveloped virus such as influenza A and HSV- 1 & 2 but not against non-enveloped viruses namely rotavirus and adenovirus^33^. A recent study also found that a combination of thymol, eucalyptol, menthol and methyl salicylate was effective against SARS-CoV-2 both *in-vivo* and *in-vitro*^28,34^. However, thymol alone showed very minimal virucidal effect (log10 0.5 to 0.75) against SARS-CoV-2 as shown in this study^28^.

Salt water gargling has long been recognised as a popular home remedy to alleviate symptoms for sore throat and colds. An on-going study is examining its effect of COVID-19 symptom relief ^37^, but besides that there is little evidence for the antiviral activity of salt water. A study by Lieleg et al. demonstrated that mucin in buffers with high salt concentration (0.3 M) increased the mucin barrier function and could blocked viral infection in vitro ^38^. Ramalingam et al. revealed that increasing concentration of sodium chloride (NaCl) enhanced the antiviral activity of epithelial cells through inhibition of RNA and DNA viral replications including human coronavirus 229E^35^. However, this study found that the NaCl solution had no virucidal activity against SARS-CoV-2, even though high concentration of NaCl (0.34 M) was used. More study should be done using various NaCl concentrations to determine the effect of NaCl against SARS-CoV-2.

This is in vitro virucidal activity of mouthwash/gargle formulations against SARS-CoV-2 suggesting that the formulations are able to reduce virus load inside the nasopharynx and oropharynx by targeting virus particles. It is emphasized that the data obtained in this study is applicable to the particular brand of mouthwash/gargle formulations; other products containing the same active ingredients would have to be verified as formulations may vary among different brands. Nonetheless, this study and other recent studies showed that most commercially available mouthwash/gargle formulations were able to inactivate SARS-CoV-2. The most effective active ingredients are povidone-iodine^17^, benzylkonium chloride, essential oils^28^, cetylperidinium chloride and hexetidine.

## Conclusions

A number of commercially available mouthwash/gargle formulations containing cetylperidinium chloride and hexetidine demonstrated potent and rapid virucidal activity of ≥ 5 log10 reduction of SARS-CoV-2 at 30 s. Chlorhexidine digluconate was slightly less effective,while salt water and thymol did not show any significant reduction in virus titre. This information gives more options for healthcare workers particularly dentists, anaesthetists, maxillofacial surgeons and otorhinolaryngologists to routinely practice pre-procedural gargling with readily available formulations. The practice of using tested mouthwash/gargle products should also be extended to high-risk groups and the general population to minimize the risk of viral transmission particularly from asymptomatic/presymptomatic carriers.

## Appendix

Raw data according to the virus CPE. Number 4 indicating of full CPE in each well and number 0 indicate as healthy cells. Each dilution has 4 replicates.

**Table:**

	Mouthwash	Contact time	Dilution (Log10)					
			1	2	3	4	5	Log10 reduction
First assay	Colgate	30	0000 0000	0000 0000	0000 0000	0000 0000	0000 0000	5 5
	Clean Dirty	60	0000 0000	0000 0000	0000 0000	0000 0000	0000 0000	5 5
	Oradex	30	4444 4444	0000 0000	0000 0000	0000 0000	0000 0000	4 4
	Clean Dirty	60	4444 4444	0000 0000	0000 0000	0000 0000	0000 0000	4 4
	Thymol	30	4444 4444	4444 4444	4444 4444	4444 4444	0044 0044	0.5 0.5
	Clean Dirty	60	4444 4444	4444 4444	4444 4444	4444 4444	0444 0044	0.75 0.5
	Bactidol	30	0000 0000	0000 0000	0000 0000	0000 0000	0000 0000	5 5
	Clean Dirty	60	0000 0000	0000 0000	0000 0000	0000 0000	0000 0000	5 5
	Salt water	30	4444 4444	4444 4444	4444 4444	4444 4444	4444 4444	0 0
	Clean Dirty	60	4444 4444	4444 4444	4444 4444	4444 4444	4444 4444	0 0
	Virus control	30	4444 4444	4444 4444	4444 4444	4444 4444	4444 4444	– –
	Clean Dirty	60	4444 4444	4444 4444	4444 4444	4444 4444	4444 4444	– –
Second assay	Colgate	30	0000 0000	0000 0000	0000 0000	0000 0000	0000 0000	5 5
	Clean Dirty	60	0000 0000	0000 0000	0000 0000	0000 0000	0000 0000	5 5
	Oradex	30	4444 4444	0000 0000	0000 0000	0000 0000	0000 0000	4 4
	Clean Dirty	60	0444 4444	0000 0000	0000 0000	0000 0000	0000 0000	4.25 4
	Thymol	30	4444 4444	4444 4444	4444 4444	4444 4444	0044 0044	0.5 0.5
	Clean Dirty	60	4444 4444	4444 4444	4444 4444	4444 4444	0444 0044	0.75 0.5
	Bactidol	30	0000 0000	0000 0000	0000 0000	0000 0000	0000 0000	5 5
	Clean Dirty	60	0000 0000	0000 0000	0000 0000	0000 0000	0000 0000	5 5
	Salt water	30	4444 4444	4444 4444	4444 4444	4444 4444	4444 4444	0 0
	Clean Dirty	60	4444 4444	4444 4444	4444 4444	4444 4444	4444 4444	0 0
	Virus control	30	4444 4444	4444 4444	4444 4444	4444 4444	4444 4444	– –
	Clean Dirty	60	4444 4444	4444 4444	4444 4444	4444 4444	4444 4444	– –
